# Supplementary material for: Mutation analysis in individual circulating tumor cells depicts intratumor heterogeneity in melanoma
Source: EMBO Mol Med. 2024 Jun 19;16(7):6. doi: 10.1038/s44321-024-00082-6 (PMC11250829; doi:10.1038/s44321-024-00082-6)

Appendix

<u>Table of contents.</u>

Appendix Table S1 ……………………………………………………………………………………………………p 2

Appendix Tables S2……………………………………………………………………………………………………p 2

Appendix Figure S1……………………………………………………………………………………………………p 3

Appendix Table S1. Lysis buffer composition.

6.6 mM Trizma-acetate pH 7.5	Carl Roth, Karlsruhe, Germany
6.6 mM Magnesium-acetate	Carl Roth, Karlsruhe, Germany
33.3 mM potassium-acetate	Carl Roth, Karlsruhe, Germany
0.44% Tween	Carl Roth, Karlsruhe, Germany
0.44% IGEPAL CA-630	Carl Roth, Karlsruhe, Germany
0.10 unit of Proteinase K	800U/ml, New England Biolabs, Ipswich, MA, US
diluted in PBS	

Appendix Table S2. PCR 1, PCR 2 and SAP preparation mixes.

**PCR#1**	µL	
water	14.04	Qiagen, Hilden, Germany
10x pcr buffer	2	Agena Bioscience, San Diego, CA, USA
mgcl2	1.6	Agena Bioscience, San Diego, CA, USA
dNTP mix	0.1	Agena Bioscience, San Diego, CA, USA
pcr primer p01	0.12	Agena Bioscience, San Diego, CA, USA
pcr enzyme	0.64	Agena Bioscience, San Diego, CA, USA
ung enzyme	0.5	Agena Bioscience, San Diego, CA, USA
Volume	19	
Lysed single cell (input)	3	
Total	22	

**PCR#2**	µL	
water	17.32	Qiagen, Hilden, Germany
10x pcr buffer	4	Agena Bioscience, San Diego, CA, USA
mgcl2	3.2	Agena Bioscience, San Diego, CA, USA
dNTP-dUTP mix	0.2	Agena Bioscience, San Diego, CA, USA
pcr primer p01	8	Agena Bioscience, San Diego, CA, USA
pcr enzyme	1.28	Agena Bioscience, San Diego, CA, USA
ung enzyme	0	Agena Bioscience, San Diego, CA, USA
Volume	34	
PCR#1 product (input)	6	
Total	40	

PCR#2 was performed in duplicate for patient samples; both reactions were pooled for analysing all variants covered by the panels.

SAP	µL	
Water	12.24	Qiagen, Hilden, Germany
SAP buffer	1.36	Agena Bioscience, San Diego, CA, USA
SAP enzyme	2.4	Agena Bioscience, San Diego, CA, USA
Volume	16	

Appendix Figure S1. Recapitulative figure for the mutations detected among the different types of samples (tissues, cfDNA, CTC) for the 33 melanoma patients at the beginning of the new line of treatment.

On the top of the panel, patient ID is indicated with the type of sample analyzed. We distinguished samples obtained before initiation of treatment from those obtained during treatment. 'Tissue UltraSEEK' stands for the analysis of gDNA from a tumor tissue sample with UltraSEEK® melanoma panel. The assays with positive values on leukocytes validation set are marked with an asterisk.

SDHD.Mut3 stands for SDHD mutation Chr11:111, 957,544 C>T.

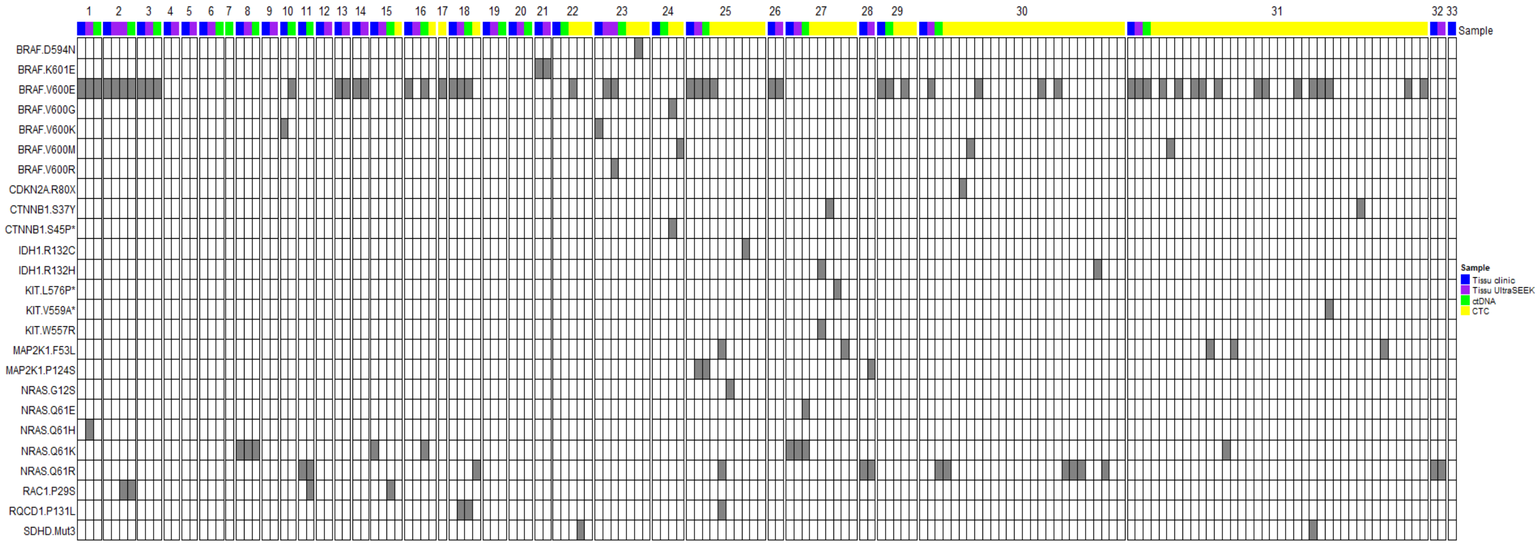

Supplement: Supplementary file 2 — Appendix [file 44321_2024_82_MOESM2_ESM.pdf]
