# Supplementary figures and images for: Mutation analysis in individual circulating tumor cells depicts intratumor heterogeneity in melanoma
Source: EMBO Mol Med. 2024 Jun 19;16(7):6. doi: 10.1038/s44321-024-00082-6 (PMC11250829; doi:10.1038/s44321-024-00082-6)

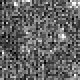

Supplement: Supplementary file 12 — Source data Fig. 2 [file 44321_2024_82_MOESM12_ESM.zip › Figure 2/Figure 2B_CD45_CD34.tiff]

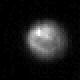

Supplement: Supplementary file 12 — Source data Fig. 2 [file 44321_2024_82_MOESM12_ESM.zip › Figure 2/Figure 2B_DAPI.tiff]

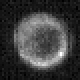

Supplement: Supplementary file 12 — Source data Fig. 2 [file 44321_2024_82_MOESM12_ESM.zip › Figure 2/Figure 2B_MEL.tiff]

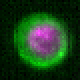

Supplement: Supplementary file 12 — Source data Fig. 2 [file 44321_2024_82_MOESM12_ESM.zip › Figure 2/Figure 2B_Merged.tiff]

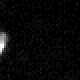

Supplement: Supplementary file 12 — Source data Fig. 2 [file 44321_2024_82_MOESM12_ESM.zip › Figure 2/Figure 2C_bottom_CD45_CD34.tiff]

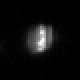

Supplement: Supplementary file 12 — Source data Fig. 2 [file 44321_2024_82_MOESM12_ESM.zip › Figure 2/Figure 2C_bottom_DAPI.tiff]

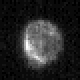

Supplement: Supplementary file 12 — Source data Fig. 2 [file 44321_2024_82_MOESM12_ESM.zip › Figure 2/Figure 2C_bottom_MEL.tiff]

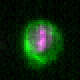

Supplement: Supplementary file 12 — Source data Fig. 2 [file 44321_2024_82_MOESM12_ESM.zip › Figure 2/Figure 2C_bottom_Merged.tiff]

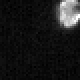

Supplement: Supplementary file 12 — Source data Fig. 2 [file 44321_2024_82_MOESM12_ESM.zip › Figure 2/Figure 2C_up_CD45_CD34.tiff]

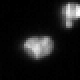

Supplement: Supplementary file 12 — Source data Fig. 2 [file 44321_2024_82_MOESM12_ESM.zip › Figure 2/Figure 2C_up_DAPI.tiff]

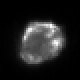

Supplement: Supplementary file 12 — Source data Fig. 2 [file 44321_2024_82_MOESM12_ESM.zip › Figure 2/Figure 2C_up_MEL.tiff]

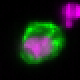

Supplement: Supplementary file 12 — Source data Fig. 2 [file 44321_2024_82_MOESM12_ESM.zip › Figure 2/Figure 2C_up_Merged.tiff]

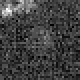

Supplement: Supplementary file 12 — Source data Fig. 2 [file 44321_2024_82_MOESM12_ESM.zip › Figure 2/Figure 2D_CD45.tiff]

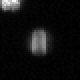

Supplement: Supplementary file 12 — Source data Fig. 2 [file 44321_2024_82_MOESM12_ESM.zip › Figure 2/Figure 2D_DAPI.tiff]

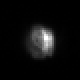

Supplement: Supplementary file 12 — Source data Fig. 2 [file 44321_2024_82_MOESM12_ESM.zip › Figure 2/Figure 2D_Keratins.tiff]

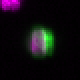

Supplement: Supplementary file 12 — Source data Fig. 2 [file 44321_2024_82_MOESM12_ESM.zip › Figure 2/Figure 2D_Merged.tiff]
