# Supplementary material for: Mutation analysis in individual circulating tumor cells depicts intratumor heterogeneity in melanoma
Source: EMBO Mol Med. 2024 Jun 19;16(7):6. doi: 10.1038/s44321-024-00082-6 (PMC11250829; doi:10.1038/s44321-024-00082-6)
Supplement: Supplementary file 17 — Expanded View Figures [file 44321_2024_82_MOESM17_ESM.pdf]

Expanded View Figures

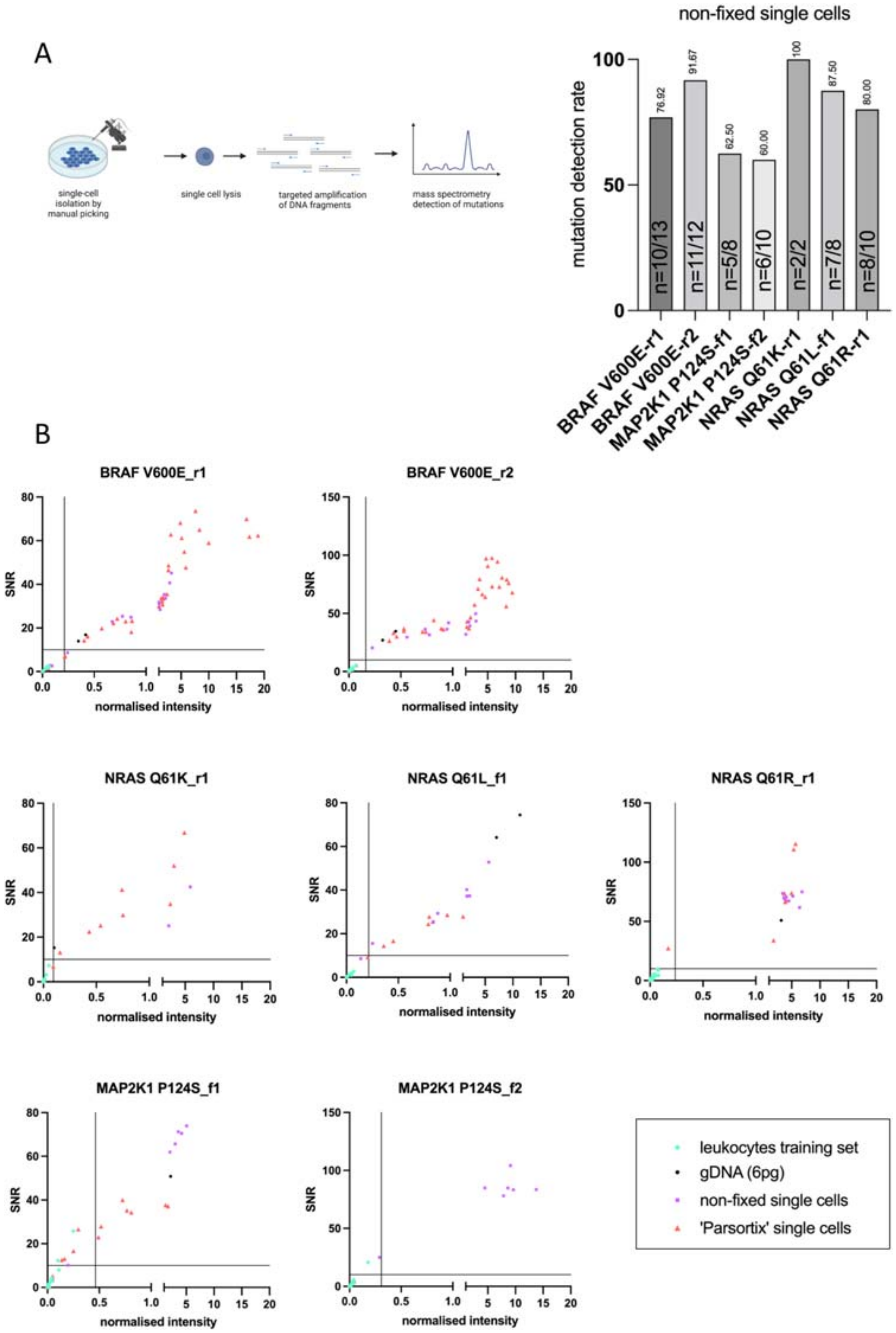

Figure EV1. Mutation detection at the single cell level with the UltraSEEK Melanoma Panel.

(**A**) The UltraSEEK® Melanoma Panel was tested on 5 mutations (certain mutations are detected by 2 different assays in the same panel) with our modified protocol on individual cells from melanoma tumor cell lines not chemically preserved. The number of cells with successful mutation detection over the number of efficiently lysed cells is mentioned. Created with BioRender.com. (**B**) Normalized intensity and signal to noise ratio (SNR) values obtained for each PCR assay for gDNA tested at 6.6 pg input in duplicate, not chemically preserved single cells and 'Parsortix' processed single cells. The horizontal line represents the threshold value of SNR (set to 10) and of the normalized intensity calculated on leukocytes. For MAP2K1 P124S_f2, only non-fixed single cells data are displayed due to the positive signal observed on one leukocyte that led to excluding this assay from further analysis. Source data are available online for this figure.

 © The Author(s)

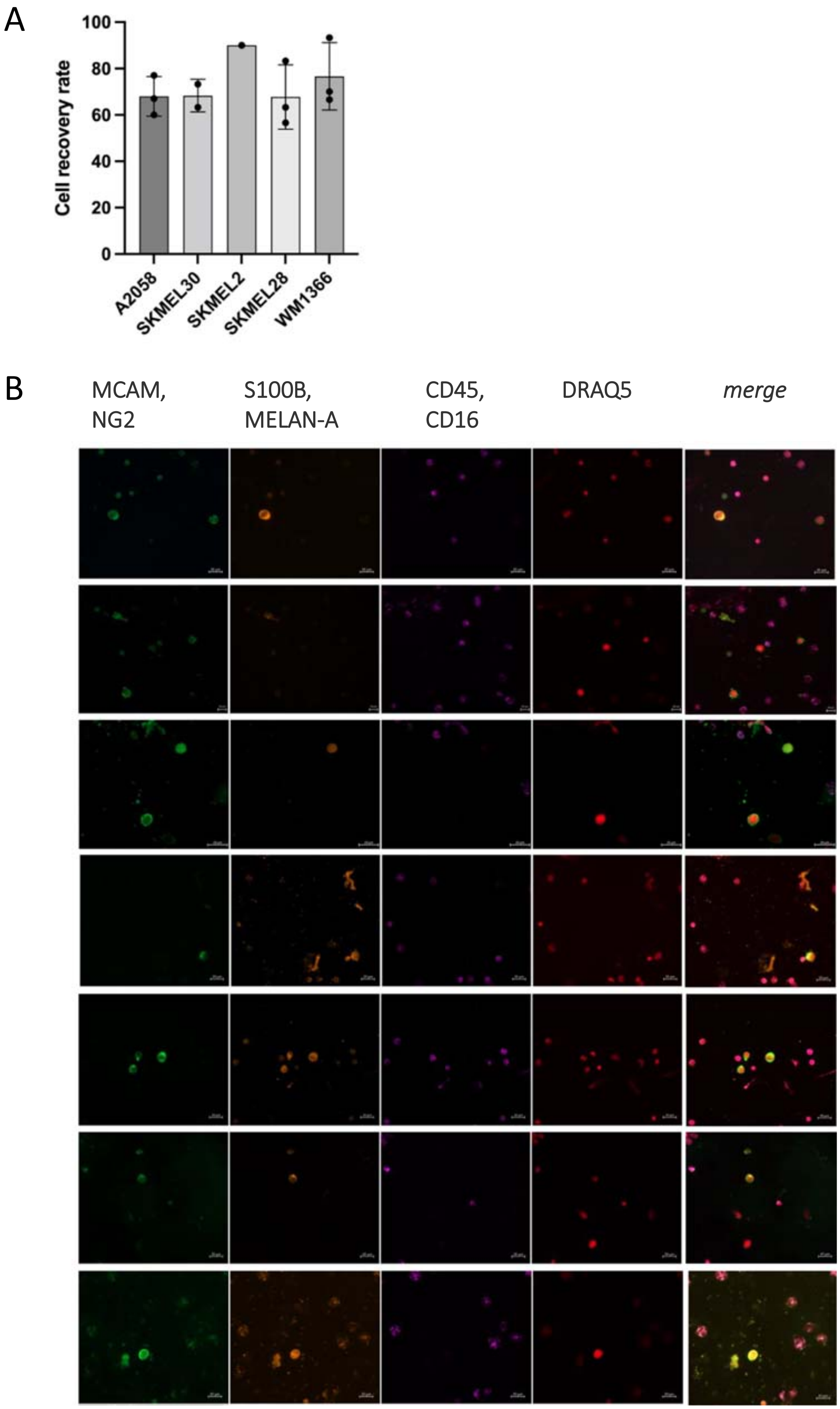
A
Cell recovery rate
100
80
60
40
20
0
A2058
SKMEL30
SKMEL2
SKMEL28
WM1366
B
MCAM, NG2
S100B, MELAN-A
CD45, CD16
DRAQ5
merge

Figure EV2. Melanoma CTC enrichment with Parsortix® workflow.

(**A**) Cell recovery rate for different melanoma cell lines. Recovery rate was calculated from spiking 30 melanoma cells into blood of healthy donors. Each dot represents one technical replicate, bar height represents the mean of recovery rate and error bars Standard Deviation. (**B**) Illustration of melanoma CTCs found in patients. CTCs were defined as nucleated elements (DRAQ5 positive) either MCAM/NG2-positive and/or Melanoma-marker/S100B-positive but CD45/CD16-negative cells. Source data are available online for this figure.

 © The Author(s)

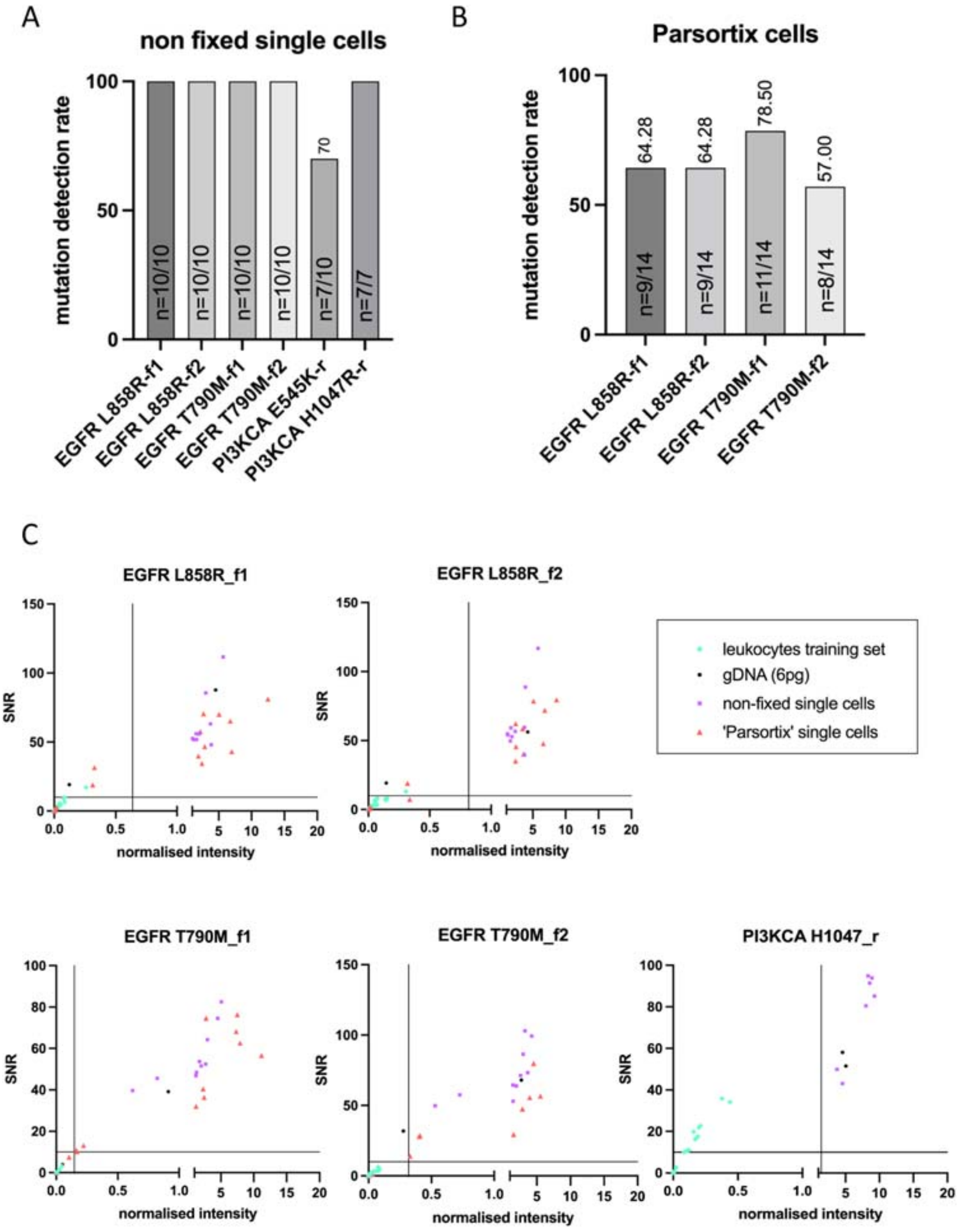

Figure EV3. Mutation detection at the single cell level with the UltraSEEK® Lung Panel.

(**A**) The UltraSEEK® Lung Panel was tested on 2 *EGFR* mutations and 2 *PI3KCA* mutations with our modified protocol on individual cells from lung (H1975) or breast tumor cell lines (MCF7 and T47D) not chemically preserved. The number of cells with successful mutation detection over the number of efficiently lysed cells is mentioned. (**B**) The UltraSEEK® Lung Panel was tested on the 2 *EGFR* mutations with our modified protocol on individual cells from lung tumor cell lines processed with Parsortix® enrichment method. The number of cells with successful mutation detection over the number of efficiently lysed cells is mentioned. (**C**) Normalized intensity and signal-to-noise ratio (SNR) values obtained for each PCR assay for gDNA tested at 6.6 pg input in duplicate, not chemically preserved single cells and 'Parsortix' processed single cells. Source data are available online for this figure.

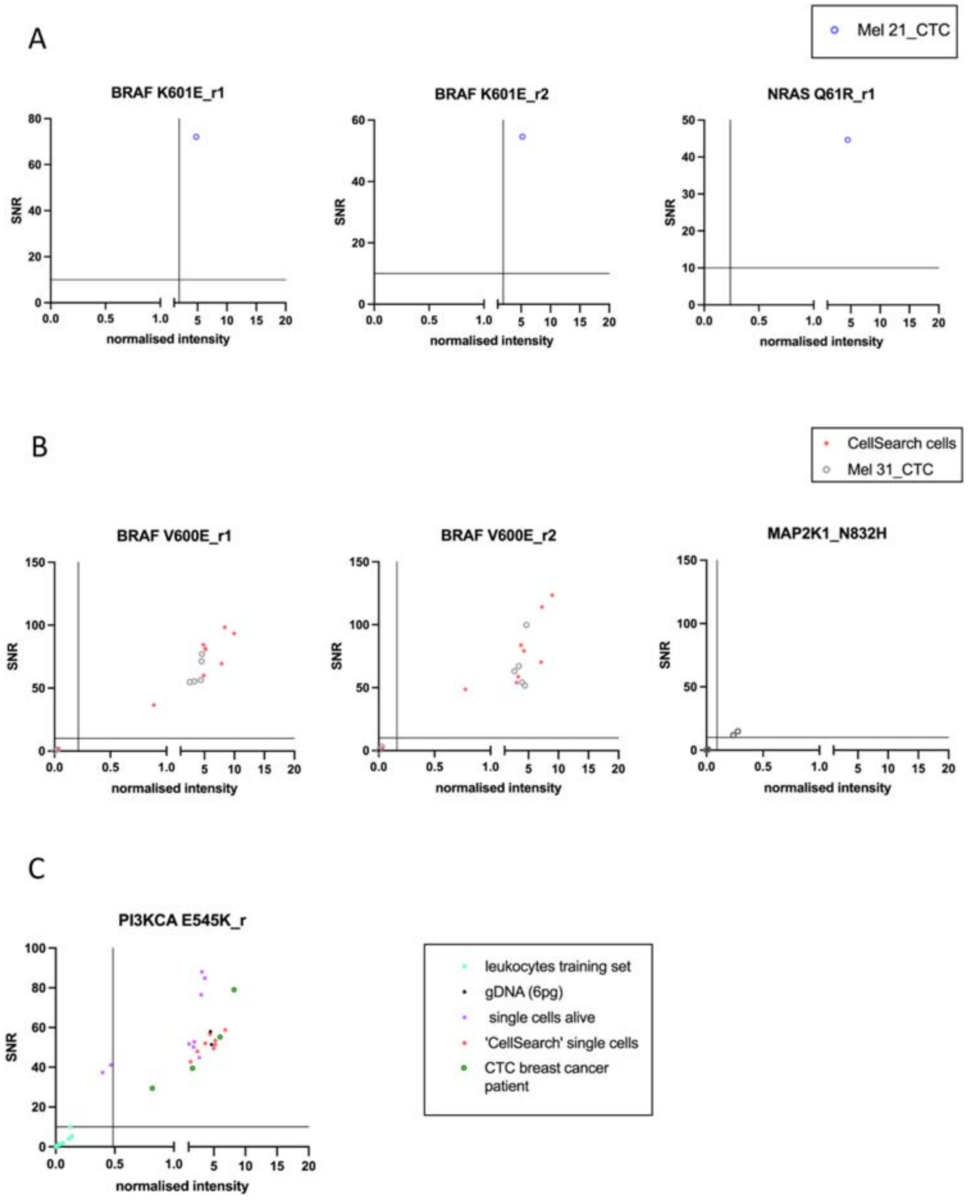

Figure EV4. Normalized intensity and SNR values for the mutations detected among CTC processed with the CellSearch® enrichment method.

For each assay, the horizontal line represents the threshold value of SNR (set to 10 according to manufacturer's recommendation) and of the normalized intensity calculated on leukocytes. (**A**) Normalized intensity and SNR values for the mutations detected among CTC from patient MEL 21. (**B**) Normalized intensity and SNR values for the mutations detected among CTCs from patient MEL 31. (**C**) Normalized intensity and SNR values for the PI3KCA E545K mutation detected among breast tumor cell lines and CTC from breast cancer patient. Source data are available online for this figure.

 © The Author(s)

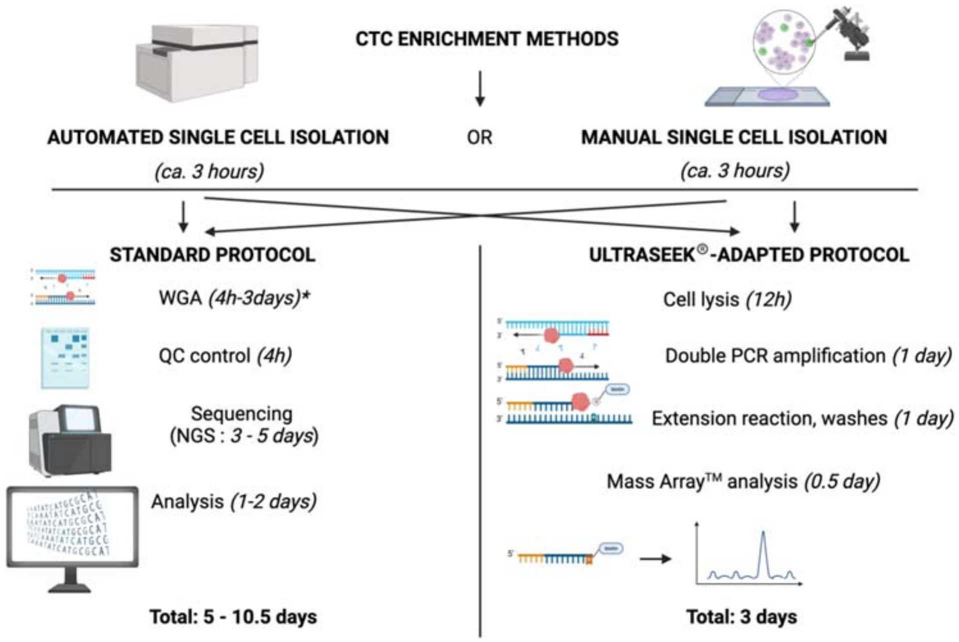

Figure EV5. Comparison of turnaround times of UltraSEEK®-adapted protocol and "standard" protocol to analyze hotspots mutations from single CTCs.

*Duration of the protocol for WGA is dependent on WGA technologies. We included cell lysis that is also necessary into WGA protocol global time. We chose to compare the UltraSEEK®-adapted protocol to WGA and next-generation sequencing (NGS) as it appears for us that NGS was the most adequate technology to analyze in parallel multiple hotspot mutations. Created using BioRender.com.
