# Supplementary material for: Mutation analysis in individual circulating tumor cells depicts intratumor heterogeneity in melanoma
Source: EMBO Mol Med. 2024 Jun 19;16(7):6. doi: 10.1038/s44321-024-00082-6 (PMC11250829; doi:10.1038/s44321-024-00082-6)
Supplement: Supplementary file 43 [file 44321_2024_82_MOESM1_ESM.docx]

Table EV 1. Tumor tissue analysis with UltraSEEK® melanoma panel for each melanoma patient.

NA stands for no assessment of the tissue.

Patient ID	Driver mutation analysis (clinical routine)				Ultraseek Melanoma analysis		
	**Tissue**	**BRAF**	**NRAS**	**KIT**	**Tissue**		
MEL1	primary tumor	V600E	WT	WT	primary tumor	BRAF V600E, >2%	-
MEL2	primary tumor	V600E	WT	WT	primary tumor	BRAF V600E, >2%	-
MEL2	sub cutaneous metastasis	V600E	-	-	sub cutaneous metastasis	BRAF V600E, >2%	RAC1 P29S, >2%
MEL3	primary tumor	V600E	WT	WT	primary tumor	BRAF V600E, >2%	-
MEL4	urethra metastasis	WT	WT	WT	urethra metastasis	-	-
MEL5	cerebral metastasis	WT	WT	WT	cerebral metastasis	-	-
MEL6	Lymph node metastasis	WT	WT	WT	Lymph node metastasis	-	-
MEL7	no tissue	-	-	-	no tissue	-	-
MEL8	primary tumor	WT	Q61K	WT	primary tumor	NRAS Q61K, >2%	-
MEL9	recidiv paranasal	WT	WT	WT	recidiv paranasal	-	-
MEL10	lung metastasis	V600K	ND	ND	no tissue	-	-
MEL11	liver metastasis	WT	Q61R	ND	no tissue	-	-
MEL12	primary tumor	WT	WT	WT	lymph node metastasis	-	-
MEL13	primary tumor	V600E	WT	WT	lymph node metastasis	BRAF V600E, >2%	-
MEL13	cerebral metastasis	V600E	WT	WT	-	-	-
MEL14	retroperitoneal metastasis	V600E	ND	ND	retroperitoneal metastasis	BRAF V600E, >2%	-
MEL15	primary tumor	WT	Q61K	ND	primary tumor	-	-
MEL16	primary tumor	V600E	ND	ND	primary tumor	-	-
MEL17	no tissue	-	-	-	no tissue	-	-
MEL18	primary tumor	V600E	WT	ND	primary tumor	BRAF V600E, >2%	RQCD1 P131L, >2%
MEL19	primary tumor	WT	WT	WT	primary tumor	-	-
MEL20	liver metastasis	WT	WT	WT	liver metastasis	-	-
MEL21	primary tumor	K601E	WT	WT	primary tumor	BRAF K601E, 1.4%	-
MEL22	liver metastasis	WT	WT	WT	no tissue	-	-
MEL23	primary tumor	V600K	ND	ND	sub cutaneous metastasis	BRAF V600E, >2%	-
MEL23	primary tumor	V600E	-	-	primary tumor	BRAF V600E, >2%	-
MEL23	lymph node metastasis	V600E	-	-	lymph node metastasis	BRAF V600E, >2%	-
MEL24	primary tumor	WT	WT	WT	no tissue	-	-
MEL25	metastasis	V600E	ND	ND	metastasis	BRAF V600E, >2%	MAP2K1 P124S, >2%
MEL26	lymph node metastasis	V600E	ND	ND	lymph node metastasis	BRAF V600E, 1%	-
MEL27	metastasis	WT	Q61K	ND	metastasis	NRAS Q61K, >2%	-
MEL28	metastasis	WT	Q61R	ND	primary tumor	NRAS Q61R, >2%	MAP2K1 P124S, >2%
MEL29	primary tumor	V600E	WT	WT	sub cutaneous metastasis	NA	-
MEL30	primary tumor	WT	WT	WT	lymph node metastasis	BRAF V600E, 0,4%	-
MEL31	subcutaneous metastasis	V600E	WT	WT	subcutaneous metastasis	BRAF V600E, >2%	-
MEL32	cutaneous metastasis	WT	Q61R	WT	cutaneous metastasis	NRAS Q61R, >2%	-
MEL33	subcutaneous metastasis	WT	WT	ND	no tissue	-	-
